# Biosolids as a Source of Antibiotic Resistance Plasmids for Commensal and Pathogenic Bacteria

**DOI:** 10.3389/fmicb.2021.606409

**Published:** 2021-04-21

**Authors:** Aaron Law, Olubunmi Solano, Celeste J. Brown, Samuel S. Hunter, Matt Fagnan, Eva M. Top, Thibault Stalder

**Affiliations:** ^1^Department of Biological Sciences, University of Idaho, Moscow, ID, United States; ^2^Department of Biological Sciences, Columbia University, New York, NY, United States; ^3^Institute for Bioinformatics and Evolutionary Studies, University of Idaho, Moscow, ID, United States; ^4^UC-Davis Genome Center, Davis, CA, United States

**Keywords:** antibiotics, antimicrobial resistance, pathogens, biosolids, plasmids

## Abstract

Antibiotic resistance (AR) is a threat to modern medicine, and plasmids are driving the global spread of AR by horizontal gene transfer across microbiomes and environments. Determining the mobile resistome responsible for this spread of AR among environments is essential in our efforts to attenuate the current crisis. Biosolids are a wastewater treatment plant (WWTP) byproduct used globally as fertilizer in agriculture. Here, we investigated the mobile resistome of biosolids that are used as fertilizer. This was done by capturing resistance plasmids that can transfer to human pathogens and commensal bacteria. We used a higher-throughput version of the exogenous plasmid isolation approach by mixing several ESKAPE pathogens and a commensal *Escherichia coli* with biosolids and screening for newly acquired resistance to about 10 antibiotics in these strains. Six unique resistance plasmids transferred to *Salmonella typhimurium*, *Klebsiella aerogenes*, and *E. coli*. All the plasmids were self-transferable and carried 3–6 antibiotic resistance genes (ARG) conferring resistance to 2–4 antibiotic classes. These plasmids-borne resistance genes were further embedded in genetic elements promoting intracellular recombination (i.e., transposons or class 1 integrons). The plasmids belonged to the broad-host-range plasmid (BHR) groups IncP-1 or PromA. Several of them were persistent in their new hosts when grown in the absence of antibiotics, suggesting that the newly acquired drug resistance traits would be sustained over time. This study highlights the role of BHRs in the spread of ARG between environmental bacteria and human pathogens and commensals, where they may persist. The work further emphasizes biosolids as potential vehicles of highly mobile plasmid-borne antibiotic resistance genes.

## Introduction

**Figure 1:**
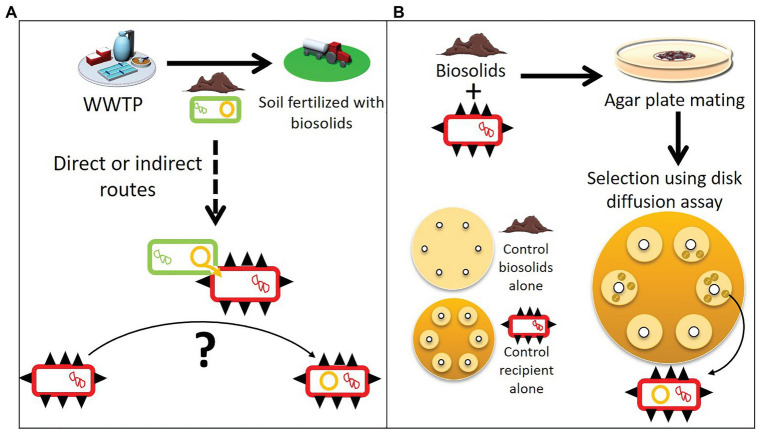
Spreading of plasmids within a mobile resistome and a modified plasmid capture method to isolate them. **(A)** Biosolids from wastewater treatment plants (WWTPs) used as agricultural soil fertilizer contain bacteria (green rectangles) with resistance plasmids (orange circles). These biosolids can spread the resistance plasmids further, and through direct or indirect routes transfer to human pathogen and commensal bacteria (red rectangles with black spikes). **(B)** To determine the mobile resistome of biosolids that can be acquired by human pathogens and commensal bacteria, we used a modified exogenous plasmid isolation approach. Biosolids were mixed individually with different ESKAPE pathogens or *E. coli* (the recipient hosts). After overnight incubation, the mixtures were spread on a large selective agar plate and stamped with about 10 antibiotic disks. Recipient hosts that acquired a new resistance plasmid from the biosolids formed colonies in the inhibition zone where the recipient alone was sensitive (“control recipient alone”). Assays with biosolids alone were also run in parallel to control for false positives (“controls biosolids alone”).

Antibiotic resistance (AR) is a threat for modern medicine across the world (WHO, 2018; Centers for Disease Control and Prevention, 2019). The spread of antibiotic resistance genes (ARG) among bacteria is largely driven by the horizontal transfer of mobile genetic elements such as plasmids (Ochman et al., 2000; Partridge et al., 2018). This pool of ARG able to transfer horizontally is often referred to as the mobile resistome. Plasmids are important vectors of horizontal gene transfer and are capable of transferring multiple ARG simultaneously, providing multidrug resistance to the recipient bacteria in one event (Ochman et al., 2000; Martínez and Baquero, 2014; Mathers et al., 2015). This is particularly true for self-transmissible plasmids that can transfer and replicate in a broad range of bacterial species (Datta and Hedges, 1972; Top et al., 1998; Jain and Srivastava, 2013). Such broad-host-range plasmids (BHR) have been shown to promote the transfer of ARG between bacteria inhabiting different environments, leading to the spread of novel genes between environments (Kruse and Sørum, 1994; Rhodes et al., 2000; da Costa et al., 2013). Plasmid-borne resistance genes are usually embedded in other mobile genetic elements such as transposons and integrons. This creates mosaic structures that facilitate change and diversity of resistance plasmids mediating AR spread (Sheppard et al., 2016). In many non-clinical environments, bacteria are reservoirs of plasmids and other mobile genetic elements that carry ARG. This poses a crucial problem, as they drive the emergence of ARG in human pathogens from unpredictable and unknown sources (Allen et al., 2010; Wright, 2010; He et al., 2020). One such hypothesized pathway is from farmland fertilized with biological products such as manure and biosolids (Figure 1A). Recognizing and understanding the sources of ARG that can be acquired by human pathogenic and commensal bacteria is important to combat the spread of ARG.

Wastewater treatment plants (WWTPs) are well-known reservoirs for AR, where many plasmids and other genetic elements involved in AR spread have been identified, such as integrons carrying ARG (Batt et al., 2007; Schlüter et al., 2007; Munir et al., 2011; Zhang et al., 2011; Brown et al., 2013; Stalder et al., 2014; Kaplan et al., 2015; McCall et al., 2016; Guo et al., 2017). Sewage sludge, a waste product of WWTPs, concentrates large amounts of the ARG from sewage (Mao et al., 2015). This sewage sludge can be treated in several ways to make a byproduct known as biosolids. Treatment involves one or a combination of techniques such as anaerobic or aerobic digestion, alkaline treatment or heat treatment, all of which are intended to reduce microbial and chemical loads in some way. Biosolids are used as fertilizer in many countries; in the United States, 60% of the 5.6 million dry tons of annually produced biosolids are used as fertilizer (National Research Council, 2002). Biosolids are not just used for agriculture, but also for other purposes such as landscaping and land reclamation in forestry (EPA, 1994). Biosolids containing ARGs are applied all over the world in numerous environments, and enrichment of soils with ARGs following the application of biosolids has been reported by several studies (Munir et al., 2011; Ross and Topp, 2015; Yang et al., 2018; Murray et al., 2019). However, there is very limited information about the resistance plasmids carried by biosolids (McCall et al., 2016).

Determining the mobile resistome in biosolids is all the more important as studies have shown that biosolid or manure application increases the relative abundance of ARG and transposable elements in soil and also on crops at harvest (Burch et al., 2014; Rahube et al., 2014a, 2016; Atidégla et al., 2016; Lau et al., 2017; Yang et al., 2018; Murray et al., 2019; Urra et al., 2019). It is thought that plasmids from bacteria in biosolids could transfer to soil bacteria. This hypothesis is reinforced by the fact that laboratory microcosms experiments showed that plasmids can transfer in soil from an introduced plasmid donor to indigenous bacteria, or to a recipient added to the soil (Weinberg and Stotzky, 1972; van Elsas et al., 1989; Hill and Top, 1998). Through direct or indirect contact, for which the routes are still not well defined, plasmid-borne resistance genes reach habitats where they can transfer to human pathogenic or commensal bacteria (Ross and Topp, 2015; Blau et al., 2018; Figure 1A).

In this study, we determined the mobile resistome of biosolids that is transferable to human pathogens and commensal bacteria. We used a modified, higher-throughput version of the exogenous plasmid isolation approach of Smalla et al. (2000), described in Figure 1B. We found that six distinct multidrug resistance plasmids transferred from biosolids to the human pathogens *Salmonella typhimurium* and *Klebsiella aerogenes*, and a commensal *Escherichia coli*. The plasmids were self-transmissible and belonged to the BHR plasmids groups IncP-1 and PromA (Van der Auwera et al., 2009). The plasmid accessory regions harboring the ARG consisted of a mosaic of multiple mobile genetic elements (transposons, IS, and integrons). Once acquired by their new host, the plasmids were able to persist without antibiotics in 20–100% of the population for at least 90 generations, showing their potential to become new reservoirs of transferable ARG. Overall the study suggests biosolids should be considered as potential vectors of ARG spread in agriculture.

## Materials and Methods

### Bacterial Strains, Media, and Biosolids

All strains and growth conditions used in this study are listed in Table 1. Unless specified, all strains were grown in Tryptic Soy agar or broth (TSA, TSB; Becton, Dickinson and Company, Franklin Lakes, NJ, United States). When media was supplemented with antibiotics, the concentrations were 50 μg ml^−1^ for kanamycin (km), 100 μg ml^−1^ for rifampicin (rif), 50 μg ml^−1^ for streptomycin (sm), 50 μg ml^−1^ for cefoxitin (fox), 10 μg ml^−1^ for tetracycline (te), 15 μg ml^−1^ for sulfamethoxazole (sx), 10 μg ml^−1^ for trimethoprim (tmp), 50 μg ml^−1^ for amoxicillin (amx), 25 μg ml^−1^ for chloramphenicol (c), and 50 μg ml^−1^ for nalidixic acid (nal). The antibiotic disks used contained an antibiotic mass per disk of 5 μg TMP, 10 μg imipenem (IPM), 10 μg meropenem (MEM), 30 μg ceftazidime (CAZ), 10 μg gentamicin (G), 10 μg colistin (CO), 30 μg TE, 20 μg amx with 10 μg clavulanic acid (AMC), 30 μg FOX, 23.75 μg sx with 1.25 μg tmp (SXT), 30 μg C, 5 μg ciprofloxacin (CIP), 15 μg erythromycin (E), and 30 μg vancomycin (V; Becton, Dickinson and Company).

**Table 1:** Bacterial strains used in this study.

Strain^a^	Incubation Temp. (°C)	Antibiotic Resistance^c^	Strain Source	Variable antibiotic disks used^d^
*Klebsiella aerogenes ATCC 13048-R* (*formerly Enterobacter aerogenes*)	30	rif, fox	ATCC 13048	C, CAZ, SXT, IPM, TMP
*Enterococcus faecalis* ATCC 19433–0052-R	37	rif, fox	ATCC 19433	C, V, CAZ, SXT, IPM, TMP
*Escherichia coli* CV601-GFP-R	37	rif, km	(Heuer et al., 2002)	AMC, FOX, CAZ, IPM, TMP
*Salmonella typhimurium* LT2 MS384-R	37	rif, sm	(Bull et al., 1997)	AMC, FOX, C, CAZ, SXT, IPM, TMP
*Shigella flexneri* ATCC 12022-R	37	rif	ATCC 12022	AMC, FOX, C, CAZ, SXT, IPM, TMP
*Shigella sonnei* ATCC 25931-R	37	rif	ATCC 25931	AMC, FOX, C, CAZ, SXT, IPM, TMP
*Staphylococcus aureus* NRS112-R	37	rif, co	BEI Resources NR-45918	FOX, C, V, SXT
*Escherichia coli* MG1655N^b^	37	nal	ATCC 47076	Not applicable

a Strains denoted with “-R” are rifampicin (rif) resistant mutants obtained by randomly selecting a resistant colony after overnight growth of the sensitive strain on an agar plate supplemented with rif.

b *Escherichia coli* MG1655N is a nalidixic acid (nal) resistant mutant of MG1655.

c Antibiotics used to select the recipient strain from biparental mating mixtures. The resistances were encoded by the chromosome. Antibiotic abbreviations are cefoxitin (fox), colistin (co), and kanamycin (km).

d All recipient strains were tested for acquisition of resistance to meropenem (MEM), ciprofloxacin (CIP), tetracycline (TE), and the other variable antibiotics listed dependent on the strain. The other abbreviations are: Chloramphenicol (C), ceftazidime (CAZ), sulfamethoxazole (SXT) with trimethoprim (TMP), imipenem (IPM), trimethoprim (TMP), vancomycin (V), amoxicillin (amx) with clavulanic acid (AMC), and FOX.

Biosolids were collected in June 2016 from one of the two WWTPs in Boise, Idaho (United States). The WWTP uses secondary treatment with ammonia removal and anaerobic digestion for 30 days at 37°C, producing Class B biosolids. Biosolid samples were kept at 4°C until further processing.

### Exogenous Plasmid Isolation

Plasmids were captured from bacteria within the biosolids through a biparental mating protocol (Smalla et al., 2000) that was modified by using multiple antibiotic disks on one large Petri dish. Recipient strains were grown overnight with antibiotic selection (Table 1, see column “Antibiotic Resistance”). About 1 ml of the culture was harvested by centrifugation at 8,000 *g* for 3 min. The pellets were washed two times through repeated resuspension in 1 ml of phosphate-buffered saline (PBS, pH 7.4) and centrifugation at 8,000 *g* for 3 min. Biosolids were suspended in sterile water at a concentration of 0.5 g ml^−1^ and vortexed thoroughly. To each of the washed bacterial samples, 500 μl of the biosolid suspension was added. The mixtures were vortexed, centrifuged as specified above, and the pellet resuspended in 100 μl of TSB that was diluted 10-fold in sterile water (0.1 TSB). Each mixture was then pipetted individually onto sterile 0.45 μm filters placed on 3-fold diluted TSB supplemented with 15 g/L of agar and 100 μg ml^−1^ cycloheximide and incubated overnight. Bacteria were then resuspended by vortexing the filters in 500 μl of PBS. Using a cotton swab, the cell suspensions were spread uniformly on Mueller Hinton Agar (MHA; Becton Dickinson) in 15-cm diameter Petri dishes supplemented with 100 μg ml^−1^ cycloheximide and antibiotics to select for the recipient. Subsequently up to 10 antibiotic disks were placed on the agar (Table 1). Cycloheximide was added to limit fungal growth. After overnight incubation, colonies growing inside the zones of inhibition of each antibiotic disk were selected as potential transconjugants, i.e., recipients that have obtained a plasmid conferring resistance to the antibiotic in the respective disk. Each transconjugant was archived in 20% glycerol stocks at −70°C. Acquisition of a plasmid by the recipient was confirmed by comparing the plasmid profiles of the transconjugant with their plasmid-free counterparts on an agarose gel (0.7% w/v) electrophoresis. The plasmid DNA extraction protocol was adapted from (Sambrook and Russell, 2001) with the following modifications: Alkaline Lysis Solution I had 50 mM Tris-Cl (pH 8.0), 2 mg ml^−1^ of lysozyme, and RNase A. The identity of the transconjugant was confirmed by amplifying the 16S rRNA genes using the universal primers 27f and 1492r (Lane, 1991) and the purified amplicons were sent to Elim Biopharm, Inc. (CA, United States) for Sanger sequencing. Details about the PCR methods can be found in the Supplementary Materials and Methods.

### Restriction Digestion to Identify Unique Plasmids

Restriction digests were carried out on plasmid DNA using BamHI and HindIII (New England Biolabs, Ipswich, MA, United States) following the protocols provided. The double digest was carried out with 50 μl DNA added to 5 μl NEBuffer 3.1, along with 45.6 Units of HindIII followed by 45 min of incubation at 37°C. Immediately after, 22.8 Units of BamHI were added and the mixture was incubated for another 15 min at 37°C. Restriction digests were analyzed by an agarose gel electrophoresis [0.7% agarose (w/v)].

### Biparental Matings to Determine Plasmid Transferability

Recipient (*E. coli* MG1655N in Table 1) and donor cells were grown overnight, followed by centrifugation of 1 ml of each culture for 3 min at 7000 *g*. The cell pellet was washed twice in PBS as in the exogenous plasmid isolation protocol, after which the cells were resuspended in 100 μl of PBS. Around 50 μl of both recipient and donor suspensions were combined, centrifuged, washed once more before resuspension in 100 μl of PBS. The donor, recipient, and mating pair were then placed individually on agar plates and incubated for 24 h before a loop-full of each was taken and resuspended in 500 μl PBS. Each cell suspension was streaked individually on a plate with selection for the recipient, a plate with selection for the presence of the plasmid, and a plate selecting for both. Successful transconjugants should grow on both and were confirmed as in the exogenous plasmid isolation protocol described above.

### Persistence Assays

Plasmid carrying strains were inoculated from glycerol stocks and incubated overnight in 5 ml of media with antibiotic selection (Table 1). Then, 4.9 μl of the cultures was transferred to 5 ml of fresh media without antibiotic selection and grown for 24 h, resulting in approximately 10 generations of growth; this procedure was repeated daily for a total of nine serial passages. Starting from the first passage and every following third day, the bacterial cultures were diluted and plated without selection to obtain distinct colonies, and then incubated overnight. The following day, 52 randomly selected colonies from the plate were replica plated onto agar supplemented with antibiotic selecting for the plasmid and onto agar with no supplements. These were incubated overnight, and the fraction of colonies growing on the selective plate to the total number of colonies grown on the non-selective plate was recorded. All assays were run with six replicates; however, for plasmids pALTS31 and pALTS33, the final time points were only represented by three replicates. When the assay indicated that a plasmid was fully persistent, verification of plasmid presence was carried out by plasmid extraction from three randomly selected colonies isolated at the end of the assay followed by an agarose gel electrophoresis as described above.

### Genome Sequencing and Assembly

Plasmids were isolated using the Qiagen Plasmid Plus Midi Kit (Qiagen, Hilden, Germany). Quality and quantity of DNA were, respectively, assessed by agarose gel electrophoresis as described above and fluorometry using the Quant-iT™ PicoGreen™ dsDNA Assay Kit (Thermo Fisher Scientific) with the TBS-380 Mini-Fluorometer (Turner BioSystems; Molecular Devices, Sunnyvale, CA, United States). Plasmids were linearized before sequencing using an enzymatic or a mechanical approach. For the enzymatic approach, we digested 15 μl of DNA sample with 135 U of nuclease S1 in the buffer provided by the manufacturer (Promega, Madison, WI, United States), and incubated the mixture at 24°C for 1 h and 60°C for 5 min. Mechanical shearing was performed using a g-tube from Covaris and less than 4 μg was centrifuged at 2,000 *g* for 60 s (Covaris Inc., Woburn, Massachusetts, United States).

DNA library preparation and sequencing were performed by the IBEST Genomics Resources Core. Short-read libraries were prepared and sequenced using an Illumina MiSeq platform (Illumina, San Diego, CA, United States) with the 300 base pair (bp) Paired-End Sample Preparation Kits (Illumina, San Diego, CA, United States). Prior to analysis, the reads were pre-processed through the read-cleaning pipeline HTStream consisting of the following steps: (1) hts_SuperDeduper to remove duplicate read pairs (possibly resulting from multi-cycle PCR reactions carried out as part of library preparation); (2) hts_SeqScreener to remove reads,^1^ which are likely to have come from PhiX and to remove sequencing adapters; and (3) hts_QWindowTrim to clean reads from low-quality bases using a sliding window approach that removes low quality ends of the reads. We obtained 93,300–150,103 paired-end reads per plasmids with more than 70% of the base quality score above 20 (Q20).

Long-read sequences were obtained with the Oxford Nanopore Technologies sequencer MinION. Library preparations were carried out using the ligation-based kit (LSK-SQK108) according to the manufacturer’s protocol. Libraries were loaded onto MinION flow cell version 9.4 (FLO-MIN106) with the Flow Cell Priming Kit EXP-LLB001 and sequenced for about ~40 h using the MinION Mk1B. Base calling and demultiplexing was done using albacore 2.0.2, yielding a total of 265,910 reads with a median size of 27,827 (min = 104 bp; max = 61,341 bp) and for which 37.7% of the bases had a quality score (Q) above 20. Demultiplexed reads were error corrected using Canu v1.6, and used in the following analysis. *De novo* assembly was performed using Unicycler version 0.4.3 by combining the data from both the Illumina short reads (see above) and the error-corrected long reads using options “-l” (Wick et al., 2017).^2^ Assembly produced a complete circular plasmid sequence for all but the plasmid pALT28. The results presented in the supplemental material suggested that in addition to the pALTS28 sequence we assembled, other structural variants of the plasmid were coexisting in the cells. Nevertheless, we confirmed that the contiguity of some of the assembled segments of pALTS28 was correct by mining the long-read sequences and using a PCR approach (see Supplementary Information).

### Annotation

Plasmids sequences were first compared to the nucleotide database of NCBI using BLASTn.^3^ Each of the plasmid nucleotide sequences showed some degree of similarity with previously sequenced and annotated plasmids from us or collaborators. These previously reported plasmid sequences were used for a first round of annotations by aligning each pair of plasmids and transferring the annotations of the genes that showed at least 98% nucleotide identity and coverage, and an intact open reading frame (ORF). This resulted in the annotation of most of the plasmid backbone genes and some genes in the accessory regions. We compared and completed the annotations of the plasmids by identifying the ORFs using Glimmer and comparing them against a homemade database composed of the RefSeq plasmid proteins using BLASTp, keeping only the annotations with again a 98% identity and coverage. Then remaining predicted ORFs not qualified by either method described above were transferred from the closely related plasmid but listed as hypothetical_proteins. Finally, IS elements were searched using ISFinder,^4^ ARG were identified and annotated using AMRFinder version 3.1.1b,^5^ and integrons were annotated by the team of INTEGRALL.^6^ Any remaining unidentified ORFs were labeled as hypothetical proteins unless the total length was less than 150 bp, in which case the ORF was removed. After removal based on length, for any set of ORFs that overlapped with one another, one of them was removed, keeping the ORF with the highest identity to a listed protein. Sequences were partially processed and analyzed using Geneious Prime (Geneious Prime® 2019.2.1). CLC sequence viewer of CLC Genomics Workbench 11.0 was used to generate plasmid mappings and the Artemis Comparison Tool (Carver et al., 2005) was used in generating plasmid alignments.^7^

The genome sequences of plasmids pATLS27, pALTS28, pALTS29, pALTS31, and pALTS33 are available in the nucleotide archives under the accession numbers MN366356-MN366361 and the gene list is available in the supplementary information (Supplementary Table S3).

### Statistics

To test if the plasmid persistence of the strains were different at the end of the persistence assay, we used a linear mixed-effect model approach using the R package nlme (Pinheiro et al., 2020). Using such a model, we nested the random factor “replicate” to account for replicated measure into the fixed factor “strain” and analyzed the variance tables for the model. We then performed a *post hoc* analysis on the model using Tukey’s test, using the function glht() of the multcomp R package (Hothorn et al., 2008).

## Results

### Capture of Antibiotic Resistance Plasmids From Biosolids

To identify the types of resistance plasmids that biosolids can successfully transfer to pathogenic or commensal bacteria, we used an exogenous plasmid isolation approach. In this biparental mating, the biosolids were the putative plasmid donors and the recipients a set of pathogenic bacterial species and a commensal *E. coli* (Table 1). Each mating mixture of biosolids and recipient was then plated to select for those recipients that acquired one or more new resistance traits using antibiotic disks. As the disks produced inhibition zones, where sensitive bacteria were unable to grow, any colony growing in these zones would indicate a recipient strain that acquired resistance to that antibiotic. Negative controls with either parental cell suspension ensured that the observed colonies were true transconjugants and not indigenous biosolids bacteria or plasmid-free recipients. The new AR phenotypes were confirmed by streaking the resulting colonies on agar plates containing both the antibiotic present in the respective disk, and the antibiotic to which the recipient was intrinsically resistant. The acquisition of a new plasmid was verified by the presence of additional plasmid band(s) on agarose gel electrophoresis when comparing plasmid-DNA extracts between the transconjugants and their respective recipient (Supplementary Figure S1). Finally, the identity of the recipient was confirmed by sequencing the 16S rRNA gene. A total of 16 plasmids were captured in *S. typhimurium*, *K. aerogenes*, and a non-pathogenic *E. coli*, while none were captured in *Enterococcus faecalis*, *Shigella flexneri*, *Shigella sonnei*, or *Staphylococcus aureus*. Of these 16 plasmids, six had a unique restriction digestion profile suggesting they were distinct (Supplementary Figure S1). One of the plasmids, pALTS27, was acquired by both *S. typhimurium* and *E. coli*. All six plasmids were transferred from the new hosts to a non-pathogenic *E. coli* in biparental matings, confirming the plasmids were self-transmissible.

### Genomic Characterization of the Antibiotic Resistance Plasmids

**Figure 2:**
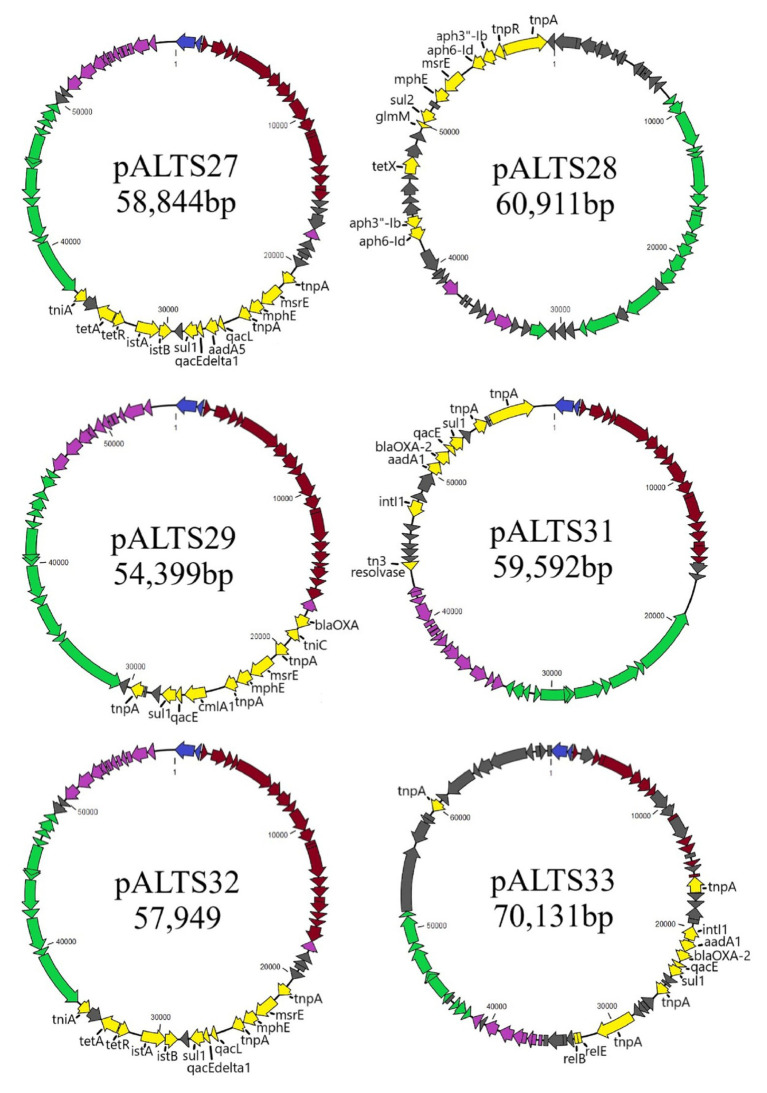
Genomic maps of the pALTS plasmids. Genes are color-coded by functional/regional categorization. All annotations are referenced in Supplementary Table S1. 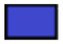 Replication, 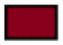 Mating Pair Formation, 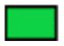 DNA Transfer, 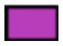 Maintenance and Control, 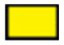 Accessory, and 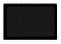 Unknown.

**Figure 3:**
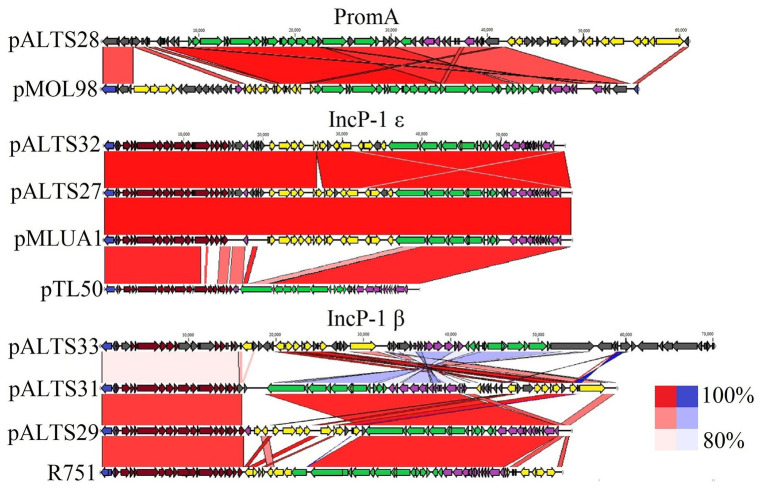
Comparative analysis of the pALTS plasmids with close representatives of their respective plasmid incompatibility group. Alignments of plasmids to representative plasmids in their group show high similarities in backbones. Red alignments represent forward alignment whereas blue alignments represent inverted alignment, and the color intensity is proportional to % similarity. Identity cutoff for visual representation was 80%. The plasmids pMLUA1 and pMOL98 are shown as close relatives of pALTS27/pALTS32 and pALTS28, respectively, while R751 and pTL50 (Shintani et al., 2020) are shown as the respective plasmid archetypes of the IncP-1β and IncP-1ɛ groups. 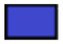 Replication, 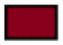 Mating Pair Formation, 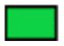 DNA Transfer, 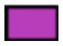 Maintenance and Control, 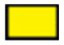 Accessory, and 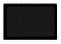 Unknown.

**Figure 4:**
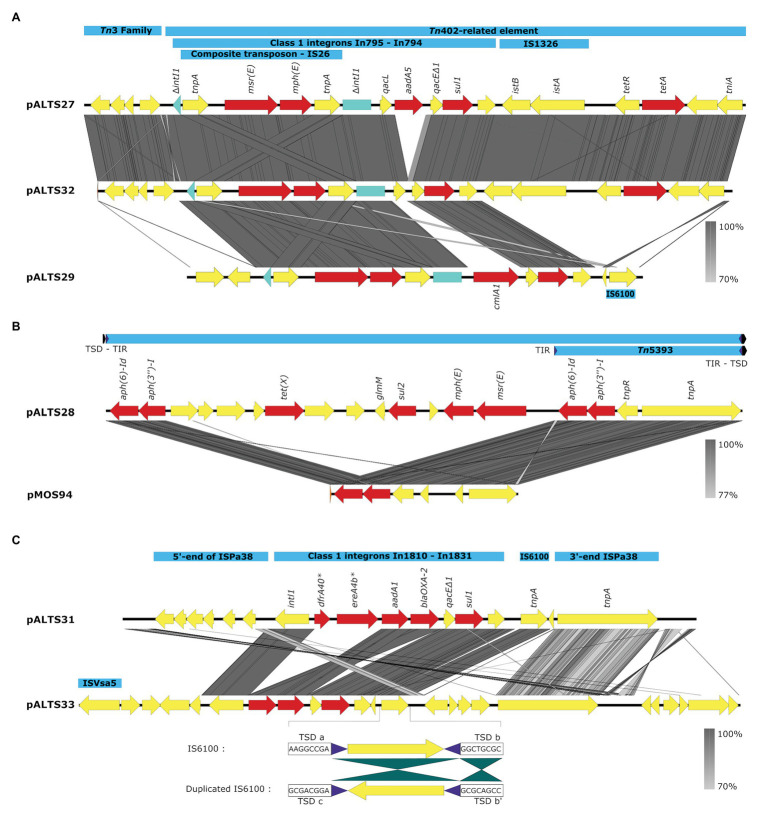
Comparative analysis of the accessory regions of the pALTS plasmids showing key mobile genetic elements in blue, ARGs in red, and other CDSs in yellow. **(A)** Comparisons of accessory regions of pALTS27, pALST32, and pALTS29. The interrupted class 1 integron integrase gene is shown in green. **(B)** Comparison of pALTS28 accessory region with its best BLASTn hit (www.ncbi.com), a mobile genetic element of the plasmid pMOS94 (accession: MK671725.1). **(C)** Comparison of accessory regions of pALTS31 and pALTS33, including details of the duplicated IS6100 in pATS33 showing the inverted conformation of the duplication and the target site duplication (TSD); b’ is the inverted TSD of b. Terminal inverted repeat (TIR) are shown by purple arrows and TSD by a black arrow. Comparative analysis was visualized using Easyfig 2.2.2 (Sullivan et al., 2011) using a predicted protein similarity threshold above 70%. *In1810 carried two putative ARG cassettes *dfr40* and *ereA4b* not identified by AMRfinder. Those two predicted proteins were 100% identical to a trimethoprim-resistant dihydrofolate reductase DfrA and an EreA family erythromycin (E) esterase in the non-redundant protein database from NCBI.

Genomic characterization of the unique plasmids by whole genome sequencing and detailed in Figure 2 showed that five belonged to the IncP-1 group of BHR plasmids, more specifically the subgroups IncP-1β and IncP-1ɛ (Table 2). The sixth plasmid, pALTS28, belonged to the more recently defined family of BHR plasmids, PromA (Van der Auwera et al., 2009), and was the only plasmid captured in *K. aerogenes*. Plasmids pATLS27 and pALTS32 showed similarity to pMLUA1 (KC964605), pALTS28 to pMOL98 (FJ666348), pALTS29 to pB3 (AJ639924), pALTS31 to pMBUI8 (KC170279), and pALTS33 to pB1 (JX469829). The two IncP-1ɛ plasmids pALTS27 and pALTS32 only differed by one resistance gene, *aadA5*, present in a class 1 integron (Figures 3, 4). As shown in Table 2, each plasmid harbored 2–5 ARG known to encode resistance to tetracyclines, aminoglycosides, macrolides, sulfonamides, chloramphenicol, trimethoprim, or β-lactams. With five different ARG, conferring resistance to four different antibiotic classes, pALTS27 and pALTS29 carried the most ARG. The sizes of the plasmids ranged from 54 to 70 kb, and the GC content of the five IncP-1 plasmids were similar and lower than that of the PromA plasmid (Table 2). A diversity of multi-drug resistance BHR plasmids present in biosolids used as fertilizer were thus able to transfer to and replicate in human pathogens.

**Table 2:** Summary of captured plasmids and their antibiotic resistance genes (ARG).

Plasmid	Accession number	Group	Length (bp)	GC Content	Resistance Genes	Integron	Capture Recipient
pALTS27	MN366356	IncP-1ɛ	58,844	59.6%	*aadA5*, *mphE*, *msrE*, *sul1*, *tetA*	In*795*	*E.c. S.t.*
pALTS28	MN366357	PromA-β	60,911	50.4%	*aph6-Id*, *aph3''-I*, *tetX*, *sul2*, *mphE*, *msrE*	NA	*K.a.*
pALTS29	MN366358	IncP-1β	54,399	62.9%	*cmlA1*, *mphE*, *msrE*, *sul1*	In*1809*	*S.t.*
pALTS31	MN366359	IncP-1β	59,592	63.8%	*aadA1*, bla_OXA-2_, *sul1*, dfr40^*^, ereA4b^*^	In*1810*	*E.c.*
pALTS32	MN366360	IncP-1ɛ	57,949	59.6%	*mphE, msrE, sul1, tetA*	In*794*	*E.c.*
pALTS33	MN366361	IncP-1β	70,131	62.6%	*aadA1*, *bla_OXA-2_*, *sul1*	In*1831*	*S.t.*

E.c., *Escherichia coli*; K.a., *Klebsiella aerogenes*; and S.t., *Salmonella typhimurium*.

* In1810 carried two putative ARG cassettes dfr40 and ereA4b not identified by AMRfinder. Those two predicted proteins were 100% identical to a trimethoprim-resistant dihydrofolate reductase DfrA and an EreA family erythromycin esterase in the non-redundant protein database from NCBI.

### Comparative Analysis of the Plasmids

There was a high degree of similarity across all the plasmids within the IncP-1 subgroups (see Figure 3). The most extreme example was pALTS27 and pALTS32, which were identical except for a single accessory gene on pALTS32 corresponding to the *aadA5* integron gene cassette of the class 1 integron array. The plasmid pALTS27 was identical to a previously described plasmid, pMLUA1 except for a single nucleotide deletion and a single nucleotide polymorphism, which could be a result of sequencing error. Plasmid pMLUA1 is an IncP-1ɛ plasmid that was previously isolated from estuarine water in Portugal (Oliveira et al., 2013).

All plasmids carried multiple transposable elements and the five IncP-1 plasmids carried at least one class 1 integron in their accessory region (Figure 4). Class 1 integrons are bacterial genetic elements capable of capturing and expressing genes embedded within cassettes. They play a major role in the global dissemination of AR by transporting and expressing ARG at the global scale (Gillings et al., 2008; Stalder et al., 2012). Here, all the class 1 integrons had the 3'-conserved sequence *qacE*delta1-*sul1-orf5* (note that *orf5* is annotated as n-acetyltransferase of unknown function). The two very similar IncP-1ɛ plasmids pALTS27 and pALTS32 and the IncP-1β plasmid pALTS29 shared the same class 1 integron integrase gene that was disrupted by the insertion of a composite transposon (IS*26*), which carried two genes conferring MLS resistance (Figure 4A). Other than this, the cassette arrays on these three plasmids were different. Since IS*26* does not transpose into specific target sites, the integration of the composite transposon in the integron integrase probably predated the gene cassette rearrangement, and the recombination event would have happened by a trans-acting integrase.

Plasmid pALTS33 was set apart from the others by an atypically large inversion encompassing both an accessory gene region and part of one of the two DNA transfer regions (Figure 3). The inversion was flanked by two identical copies of IS6100 and IS of the IS*6*/IS*26* family present in an inverted orientation (Figure 4C). These duplicated IS*6*/IS*26* family elements shared a target site duplication (TSD) that was also in an inverted orientation (Figure 4C). This conformation indicates that the large inversion was the product of an intramolecular transposition of the IS element. Indeed, when the 3'-OH groups resulting from the cleavage at both terminal inverted repeat (TIR) of the IS attack the target site on the opposite strand of the same molecule, the target site and the DNA bracketed by the original IS and the new copy become inverted (He et al., 2016).

Plasmid pALTS28 was categorized as a PromA-β plasmid. Even though there are only a few other PromA plasmid sequences to compare to, the similarity between its presumed backbone and that of pMOL98 was quite high (Figure 3). Unlike pMOL98, which was a cryptic plasmid without native resistance genes (Van der Auwera et al., 2009), pALTS28 contained the Tn*5393* element of 5,470-bp carrying the genes *aph(3'')Ib* and *aph(6)-Id*, encoding resistance to sm. Interestingly, these two resistance genes and the left TIR of Tn*5393* were duplicated further upstream in the plasmid (Figure 4B). This duplication of the left TIR and the single right TIR bracketed a 18,531-bp fragment that has the potential to transpose and is composed of 12 additional ORFs, including the resistance genes *sul2*, *tet(X)*, *mph(E)*, and *msr(E)*. A TSD typically frames transposons and is the product of the transposition event. Screening for such a site identified a direct repeat of four bases (TATT) framing this large 18,531-bp version of the transposon, but no direct repeat framing the Tn*5393* could be detected. These results suggest that the larger version of the transposon transposed into pALTS28, resulting in the acquisition of multiple ARG. As far as, we know pALTS28 is the first plasmid in the PromA group reported to confer AR.

### Plasmid Persistence

**Figure 5:**
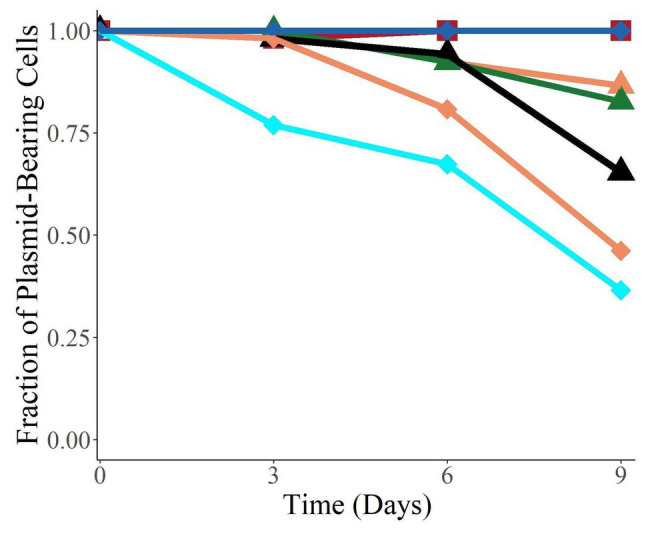
The plasmids were able to persist in their new host over 9 days without selection. Species are coded by shape, and plasmids by color. The proportion of plasmid-carrying cells reported is the median of six replicate cultures. On day 9, the data for *E. coli* (pALTS31) and *S. typhimurium* (pALTS33) only represent triplicates. 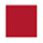 *Klebsiella aerogenes* (pALTS28), 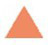 *E. coli* (pALTS27), 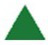 *E. coli* (pALTS31), 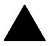 *E. coli* (pALTS32), 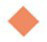 *S. typhimurium* (pALTS27), 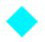 *S. typhimurium* (pALTS29), and 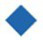 *S. typhimurium* (pALTS33).

To investigate whether the plasmids were able to persist in their new hosts in the absence of antibiotic selection once established, we measured the fraction of bacteria retaining the plasmids when the populations were grown without antibiotics for 9 days (Figure 5). All the plasmids were found to persist in their new hosts to various degrees, and four of the six were still present in over 50% of their population after 9 days (corresponding to about 90 generations). Interestingly, we observed a large variation in persistence, with plasmids pALTS28 and pALTS33 showing 100% persistence and pALTS29 only roughly 20%. Since plasmid presence is inferred by resistance to an antibiotic encoded by one of the plasmid resistance genes, integration of that gene or the entire plasmid into the chromosome cannot be excluded. Therefore, we verified and confirmed the physical presence of both plasmids in the resistant cells at the end of the assay for both *K. aerogenes* (pALTS28) and *S. typhimurium* (pALTS33; Supplementary Figure S3). This confirmed that the plasmids were still present, but pALTS28 showed a different plasmid band migration pattern with multiple bands higher in the agarose gel after electrophoresis (Supplementary Figure S3). This suggests that the plasmid conformation was altered, making it impossible to conclude that the wild-type pALTS28 is truly highly persistent and not a derivative of this plasmid. It is noteworthy but not surprising that pALTS27 displayed different persistence in two different hosts after 9 days of growth without selection, with much higher persistence in *E. coli* than in *S. typhimurium* (orange triangle and diamond in Figure 5, *p* < 0.01).

## Discussion

The spread of AR is a threat to global health. Horizontal gene transfer facilitates the transit of ARG not only within microbiomes but also between microbiomes of different habitats, to eventually end up in human pathogens and commensals. Defining the mobile pool of ARG, called the mobile resistome, has become critical to understanding these routes of spread of AR on a global scale (Gaze et al., 2013; Blau et al., 2018; Lanza et al., 2018; Stalder et al., 2019).

The exogenous plasmid isolation approach is a valuable tool to capture plasmids that mediate resistance in various environments (Smalla et al., 2015). It was first developed to capture mercury resistance plasmids from the biofilms on river rock (Bale et al., 1987). It removes the need to culture the original plasmid host, many of which may be difficult to grow in the laboratory (Staley and Konopka, 1985). Its main advantage compared to most current metagenomic approaches is that it identifies those plasmids that can actively transfer from indigenous bacteria by conjugation. Indeed, captured AR plasmids represent the mobile ARG that are in viable bacterial populations, in contrast to a metagenomics approach, which may also detect DNA from non-viable or lysed cells, or non-conjugative plasmids. Moreover, having the captured resistance plasmids in a known bacterial strain separated from other putative co-residing plasmids, allows a more detailed plasmid characterization such as the phenotypes they encode and their persistence and transferability. In this study, we used exogenous plasmid isolation to identify those AR genes in biosolids that are readily available to human commensals and pathogens by horizontal gene transfer. Specifically, we used a set of clinically relevant hosts belonging to the ESKAPE pathogens (Rice, 2008) as recipients and adapted the method by using an antibiotic disk assay, which facilitates a higher-throughput screening of multiple AR traits, including resistance to so-called last-resort antibiotics of clinical importance such as carbapenems and colistin. This adapted version of the exogenous plasmid isolation method allowed us to determine the mobile resistome in biosolids.

We focused on biosolids used as agricultural fertilizers as they are an important byproduct of wastewater treatment that could spread AR from human origin to the environment, and eventually back to human settings, for example, *via* the food chain or water (Pruden, 2014; Murray et al., 2019). We specifically tested class B biosolids. Class A and B biosolids differ by the amount of fecal coliform and pathogens that can be detected after various treatments, with the requirement for class A being more stringent than for class B, resulting in different handling regulations. While these regulations may be sufficient to reduce the hazards linked to direct contact with biosolids, they have not considered the potential impact on AR spread. In that regard, fertilization with class A biosolids was shown to present a lower risk of ARG dissemination from human origin to agricultural soils than class B (Murray et al., 2019). In our study, biosolids were obtained after anaerobic digestion, a process previously found to diminish the amounts of some ARG and class 1 integrons detected in metagenomic DNA extracts (Ghosh et al., 2009; Burch et al., 2014, 2017). Despite this, we showed that some ARG and genetic elements remaining in the biosolids microbiome are on plasmids that can transfer directly to pathogens. Fortunately, we did not capture plasmid-mediated resistance to any of the last-resort antibiotics. Nevertheless, we cannot rule out their presence as they could have been carried on other plasmids that were unable to transfer to or replicate in our recipients under our laboratory conditions. Overall, the presence of a mobile resistome in class B biosolids should be considered when these biosolids are used as fertilizers on agricultural soils.

Plasmids in WWTPs typically represent many different incompatibility groups (Schlüter et al., 2008; Rahube et al., 2014b) including those of the IncP-1 group (Schlüter et al., 2007; Bahl et al., 2009). Here too, five of the six plasmids, we captured from biosolids belonged to the IncP-1 group. The three IncP-1β plasmids showed high similarity to the previously described IncP-1β plasmids pB3, pMBUI8, and pRSB222 (Heuer et al., 2004; Brown et al., 2013; Sen et al., 2013), of which two were isolated from WWTPs, (Heuer et al., 2004; Sen et al., 2013) and one from a creek in Idaho receiving agricultural runoff (Brown et al., 2013). The two other plasmids were IncP-1ɛ plasmids that were almost identical to pMLUA1 isolated from estuarine water in Portugal (Oliveira et al., 2013), a location quite far away from Idaho. Given that mutations are not uncommon in large bacterial populations and that the accessory regions of these plasmids contained multiple transposable elements, we would expect some degree of genetic variation between plasmids isolated in different parts of the world (Yi et al., 2010). Similarly, (Petersen et al., 2019) found 100% identical plasmids among multiple distinct genera of *Rhodobacteriaceae* isolated around the world. Such a high degree of conservation around the globe among plasmids may not be new but implies some mechanisms of conservation that should be investigated. One possible explanation is the recently defined “segregational drift,” extending the time to fixation for mutations on multi-copy plasmids relative to chromosomal mutations (Ilhan et al., 2019). Our data emphasize that the resistance plasmids remaining in class B biosolids applied on agricultural lands are very similar to the ones found before treatment in the WWTP and downstream environments. Future studies should determine which bacteria are carrying these plasmids and whether plasmids transfer between hosts during the wastewater treatment and sewage sludge treatment process.

Both the IncP-1 and PromA incompatibility groups are BHR groups, but in contrast to IncP-1 plasmids very few PromA plasmids have been identified so far (Ito and Iizuka, 1971; van Elsas et al., 1998; Schneiker et al., 2001; Tauch et al., 2002; Gstalder et al., 2003; Mela et al., 2008; Van der Auwera et al., 2009; Li et al., 2014; Dias et al., 2018; Yanagiya et al., 2018; Werner et al., 2020). PromA plasmids are known for their BHR and their ability to (retro)-mobilize other less or non-transmissible plasmids within the bacterial community (van Elsas et al., 1998). Mobilizing plasmids further increase the flow of plasmid-borne AR in bacterial communities (Heuer and Smalla, 2012). The previously identified PromA plasmids were isolated from various soils, crop-related environments, manure, and wastewater, but none of them carried ARG. Conferring resistance to antibiotics is thus a new feature found on PromA plasmids, which up to now were either cryptic, or involved in metal resistance or herbicide degradation. Our data support the idea that BHR plasmids are important drivers of the mobilization of the resistome in wastewater environments and showed that they can find their way to human pathogens.

The six BHR plasmids that were captured in human commensal or pathogenic bacteria were moderately to very persistent in these hosts in the absence of antibiotics. This is disconcerting as it means these plasmids could persist for long periods of time in pathogens or commensals. The rather high but variable persistence of these plasmids in their new hosts is in line with previous reports on IncP-1 plasmids (Thomas and Helinski, 1989; De Gelder et al., 2007). The lack of any detectable plasmid loss over 90 generations for two of the plasmid-pathogen pairs is also consistent with these previous studies, yet it contrasts with the paradigm that because plasmids are often costly they should be lost from populations without selection (Harrison and Brockhurst, 2012; San Millan and MacLean, 2017). However, some IncP-1 plasmids encode post-segregational killing systems that inhibit growth of plasmid-free segregants (Easter et al., 1997). Moreover, most of the IncP-1 plasmid backbone genes are silent, thus minimizing the plasmid’s cost to the host past the first transcriptional outburst after acquisition (Fernandez-Lopez et al., 2014). Despite showing such “well-adapted behavior,” IncP-1 plasmids do not typically carry the resistances found in clinical settings and are not often found in human pathogens but have been mostly isolated from environmental and farm settings (Popowska and Krawczyk-Balska, 2013). However, they were recently found to be involved in the proliferation of the ARG *mcr-1* causing resistance to the last-resort antibiotic colistin (Zhao et al., 2017).

In conclusion, the transfer of the resistome of biosolids to human pathogens and commensals was mediated by BHR plasmids known to cross species barriers. Given the frequent use of these biosolids as fertilizer, their ability to actively transfer resistance genes to pathogens by means of these BHR plasmids is of concern. Since some of these plasmids can also be maintained without selection in human pathogens, their role in the spread of AR from environment to clinic needs to be further investigated.

## Data Availability Statement

The original contributions presented in the study are included in the article/Supplementary Material, further inquiries can be directed to the corresponding author/s.

## Author Contributions

TS and ET conceived the study. AL, OS, and TS collected the data. AL and TS analyzed and interpreted the data, and wrote the first draft of manuscript. CB helped to annotate the plasmids. SH and MF performed the sequencing. ET provided thorough critical revisions. All authors contributed to the article and approved the submitted version.

### Conflict of Interest

The authors declare that the research was conducted in the absence of any commercial or financial relationships that could be construed as a potential conflict of interest.

## Footnotes

^1^https://github.com/s4hts/HTStream

^2^https://github.com/rrwick/Unicycler

^3^https://www.ncbi.nlm.nih.gov/

^4^https://www-is.biotoul.fr

^5^https://github.com/ncbi/amr

^6^http://integrall.bio.ua.pt/

^7^https://www.qiagenbioinformatics.com/
