# Supplementary material for: Biosolids as a Source of Antibiotic Resistance Plasmids for Commensal and Pathogenic Bacteria
Source: Front Microbiol. 2021 Apr 21;12:606409. doi: 10.3389/fmicb.2021.606409 (PMC8098119; doi:10.3389/fmicb.2021.606409)
Supplement: Supplementary file 1 [file Data_Sheet_1.PDF]

SUPPLEMENTARY MATERIAL

Supplemental data

The following section details how we confirmed the assembled sequence of pALTS28.

The hybrid assembly of pALTS28, assembled with a combination of Illumina short-reads and Oxford Nanopore Sequencing (ONT) long-reads using Unicycler version 0.4.3 (Wick et al., 2017), produced 25 contigs. Two large contigs of 46,661 bp and 11,967 had a relative depth of 53.2x and 40.33x, respectively, and were not mapping to the host chromosome (*E. coli* Escherichia coli MG1655N), suggesting they corresponded to the plasmid sequence. All the other contigs had a relative depth <1.42x and mapped to the host chromosome (from here and below we used Geneious Prime mapper to map long-reads and contigs (Geneious Prime® 2019.2.1). Note that we carried out the sequencing on plasmid DNA extract, which explains why the amount of chromosome sequence was minor.

We then looked at the output assembly of the short-read sequences obtained with Spades 3.9.1 (Nurk et al., 2013). One contig of 61,462 bp had a read coverage value of 216x. In contrast, all the other assembled contigs had read coverage values <6x, suggesting the contig of 61,462 bp corresponded to the plasmid pALTS28. This contig had two 551-bp identical ends, indicating a circular sequence. We assumed this sequence of 60,911pb after circularization was the sequence of the plasmid pALTS28. Both contigs assembled by Unicycler and described above mapped to the manually closed sequence. We then analyzed the profile of the read depth coverage by mapping the short-read sequences used for the assembly back to the assembled plasmid using Bowtie2 (Langmead and Salzberg, 2012). This profile showed substantial variation in read-depth over several segments, as shown in Figure S2. These variations were in the order of 2-fold relative to the rest of the sequence, suggesting the presence of structural variation (SV), but we did not find evidence of those SV in this segment in our sequencing data. However, we confirmed that the contiguity of some of the assembled segments of pALTS28 was correct by mining the long-read sequences and using a PCR approach (see Supplementary Materials and Methods below, the Tables S1 and S2).

Mining the long-read sequences: we searched in the longest reads from the ONT long-read sequencing to see if they were matching the sequence of pALTS28. We found that all the top six longest reads, ranging from 59,856 to 60,110 bp, mapped to the 60,911pb pALTS28 sequence we assembled.

PCR junctions: we use a PCR strategy by designing primers framing all the junctions where we observed large variation in read depth suggesting potential structural variants (see Supplementary Material and Methods and Figure S2). The results of this PCR approach are presented in Table S2. All the amplified sections had the expected size amplicons given the sequence of pALTS28 we assembled and did not show any other product. These findings indicate that the section shown in Figure S2 was properly assembled despite the wide variation in read-depth coverage and suggest that the plasmid sequence of pALTS28 was correctly assembled.

Supplementary Materials & Methods

PCR. Depending on the length of the sequence, either the PCR Master Mix (2X) or Phusion® High-Fidelity DNA Polymerase mix were used based upon the protocol recommendations of the supplier (Thermo Fisher Scientific). Primer sequences are available in Table S2. In all cases, a final primer concentration of 0.5 µM was used. For 16S rRNA gene amplification, the thermocycler was programmed to run one cycle at 94° C for five minutes followed by 30 cycles of 94° C for one minute, 56° C for one minute, and 72° C for one minute, and ended with one cycle of 72° C for 5 min. For the amplifications used to verify the junctions of pALTS28, the thermocycler ran one cycle at 95° C for three minutes followed by 35 cycles of 95° C for 30 seconds, 60° C for 30 seconds, and 72° C for 1 min 30 sec, and ended with one cycle of 72° C for 5 min. The reactions done with the Phusion® High-Fidelity DNA Polymerase were run for one cycle at 98° C for 30 seconds followed by 35 cycles of 98° C for 10 seconds, 60° C for 30 seconds, and 72° C for various times depending on the primer combination (see Table S2), and ended with one cycle of 72° C for 10 min. All PCR products were confirmed through gel electrophoresis.

Supplementary Figures

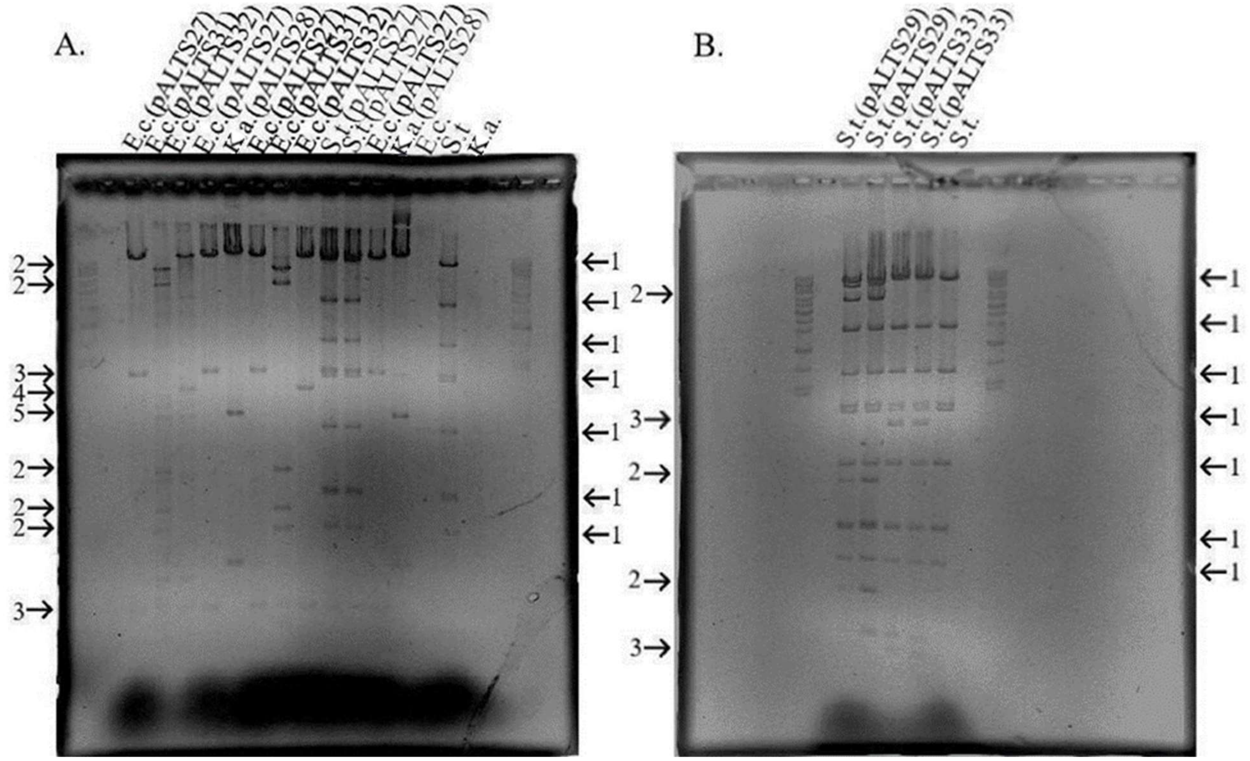

Figure S1: Agarose gel electrophoresis of plasmid DNA from initial transconjugants after plasmid capture, digested with BamHI and HindIII. Unique banding patterns were used to identify unique plasmids for further investigation. Restriction digest lanes are bordered by the Invitrogen High Molecular weight DNA Marker (40ng per lane). A) Arrow 1 points to the native plasmid in *S. typhimurium*; arrows 2-5 point to plasmids pALTS31, pALTS27, pALTS32, and pALTS28, respectively. B) Arrow 1 points to the native plasmid in *S. typhimurium;* arrows 2 and 3 point to plasmids pALTS29 and pALTS33 respectively. E.c, K.a, S.t. refer to the recipient strain *E. coli*, *K. aerogenes,* and *S. typhimurium* respectively. High Molecular Weight DNA Marker from top to bottom in base pairs were: 48,502, 38,416, 33,498, 29,942, 24,776, 22,621, 19,399, 17,057, 15,004, 12,220, 10,086, 8612, 8271.

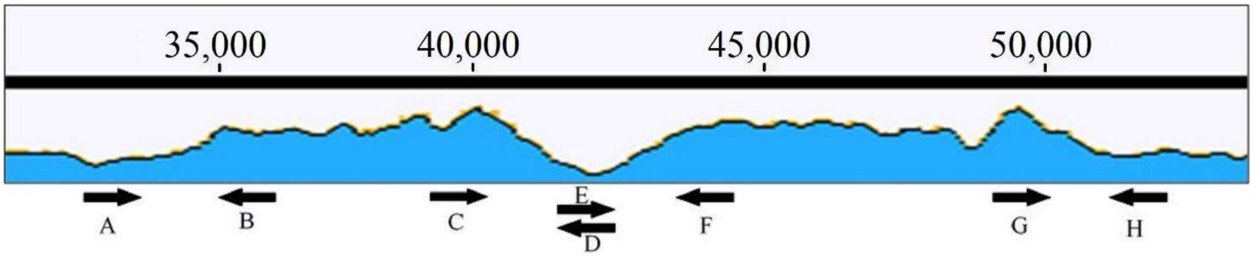

Figure S2: Profile of the read-depth coverage over a segment of pALTS28 with significant variations. The read-depth is shown by the blue shade, and the arrows indicate the location of the primers. Primers target changes in read-depth coverage to test for the existence of each junction as assembly suggested multiple possible conformations.

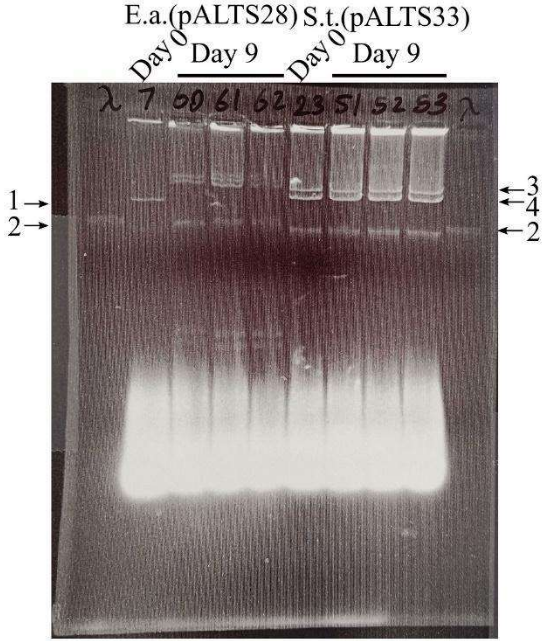

Figure S3: Agarose gel electrophoresis of plasmid DNA extracts from K.a. (pALT28) and S.t. (pALTS33) before ("Day 0") and at the end ("Day 9") of the persistence assay. Three randomly picked isolates from the end of the persistence assay were tested for each strain. Arrow 1 points to pALTS28 in line 7; arrow 2 points to the putative residual chromosomal DNA in all the lanes co-extracted during the plasmid isolation; arrow 3 points to the 90-kb native plasmid pSLT in *S.t*; arrow 4 points to pALTS33. All the extracts are framed at each end of the gel by 100 ng of linearized Lambda DNA (48.5 kb). Note that the RNAse treatment was not performed, resulting in a large amount of RNA in the extract (large bright smear) potentially covering any low molecular weight DNA.

Supplementary Tables

Table S1: Primer Sequences.

Identification	Sequence 5' - 3'
27f	AGAGTTTGATCMTGGCTCAG
1492r	TACGGYTACCTTGTTACGACTT
1847 (A)	CGGATATCGGAGGCCTCAAC
1848 (B)	CCGTTCGGAAAGTCAATTGCT
1849 (C)	TGCTCCTGGGTCTGTTTGAC
1850 (D)	TATATGCTTGAACCCGCGCT
1851 (E)	GAGGTCGATCACATCTGCCC
1852 (F)	CCTCGATCAAGGGGCATTTCTA
1853 (G)	CCCGCTGGCCTTTATCATTATG
1854 (H)	GAGAAGAGGAGCAACGCGAT

The primers 1847(A)-1852(F) correspond to those in Table S2 and Figure S2.

Table S2: PCR results to verify the contiguity of the segments of pALTS28 with differential read-depth coverage.

Primer Combination	Expected Result	Observed Result	Interpretation	PCR Extension Time at 72° C
A-B	350 bp	≈350 bp	The left-side section: A-B exists	1 min 30 sec
C-D	336 bp	≈336 bp	The left-middle section: C-D exists	1 min 30 sec
E-F	850 bp	≈850 bp	The right-middle section: E-F exists	1 min 30 sec
G-H	348 bp	≈348 bp	The right-side section: G-H exists	1 min 30 sec
C-F	2112 bp	≈2112 bp	The central section: C-F exists	2 minutes
G-B	No Amplification	No Amplification	Zone doesn't have a secondary independent circular form	1 min 30 sec
A-H	9072 bp	≈9072 bp	The full segment exists with all intermediate junctions	5 minutes

Mapping arrangement of primers is shown in Figure S2

Table S3: Plasmid Annotations.

Gene	Min.	Max	Direction	Source	Identity	Plasmid
pALTS27:						
trfA	1	1224	reverse	KC964605	100.00%	pALTS27
ssb	1275	1616	reverse	KC964605	100.00%	pALTS27
trbA	1728	2102	forward	KC964605	100.00%	pALTS27
trbB	2392	3354	forward	KC964605	100.00%	pALTS27
trbC	3368	3832	forward	KC964605	100.00%	pALTS27
trbD	3836	4147	forward	KC964605	100.00%	pALTS27
trbE	4144	6702	forward	KC964605	100.00%	pALTS27
trbF	6699	7472	forward	KC964605	100.00%	pALTS27
trbG	7490	8383	forward	KC964605	100.00%	pALTS27
trbH	8386	8841	forward	KC964605	100.00%	pALTS27
trbI	8845	10248	forward	KC964605	100.00%	pALTS27
trbJ	10267	11037	forward	KC964605	100.00%	pALTS27
trbK	11047	11220	forward	KC964605	100.00%	pALTS27
trbK	11217	11432	forward	KC964605	100.00%	pALTS27
trbL	11444	13360	forward	KC964605	100.00%	pALTS27
trbM	13396	13983	forward	KC964605	100.00%	pALTS27
trbN	14000	14629	forward	KC964605	100.00%	pALTS27
trbO	14658	14927	forward	KC964605	100.00%	pALTS27
trbP	14924	15622	forward	KC964605	100.00%	pALTS27
upf30.5	15637	16068	forward	KC964605	100.00%	pALTS27
hypothetical_protein	16111	16524	forward	KC964605	100.00%	pALTS27
hypothetical_protein	16550	17518	forward	KC964605	100.00%	pALTS27
parA	17501	18106	reverse	KC964605	100.00%	pALTS27
hypothetical_protein	18282	18818	reverse	KC964605	100.00%	pALTS27
hypothetical_protein	18830	19225	reverse	KC964605	100.00%	pALTS27
hypothetical_protein	19222	19473	reverse	KC964605	100.00%	pALTS27
hypothetical_protein	19655	20221	forward	KC964605	100.00%	pALTS27
intI1	20569	20784	forward	KC964605	100.00%	pALTS27
tnpA	20844	21560	forward	KC964605	100.00%	pALTS27
msr(E)	22010	23485	forward	KC964605	100.00%	pALTS27
mph(E)	23541	24425	forward	KC964605	100.00%	pALTS27
tnpA	24497	25213	forward	KC964605	100.00%	pALTS27
intI1	25274	26063	forward	KC964605	100.00%	pALTS27
qacL	26314	26646	forward	KC964605	100.00%	pALTS27
aadA5	26720	27508	forward	KC964605	100.00%	pALTS27
qacEdelta1	27714	28061	forward	KC964605	100.00%	pALTS27
sul1	28055	28894	forward	KC964605	100.00%	pALTS27
hypothetical_protein	29022	29522	forward	KC964605	100.00%	pALTS27
istB	29698	30480	reverse	KC964605	100.00%	pALTS27
istA	30470	31984	reverse	KC964605	100.00%	pALTS27

tetR	32826	33503	reverse	KC964605	100.00%	pALTS27
tetA	33582	34781	forward	KC964605	100.00%	pALTS27
hypothetical_protein	34813	35673	reverse	KC964605	100.00%	pALTS27
tniA	35658	36365	reverse	KC964605	100.00%	pALTS27
traC	36664	40371	reverse	KC964605	100.00%	pALTS27
traD	40375	40758	reverse	KC964605	100.00%	pALTS27
traE	40763	42838	reverse	KC964605	100.00%	pALTS27
traF	42853	43389	reverse	KC964605	100.00%	pALTS27
traG	43386	45290	reverse	KC964605	100.00%	pALTS27
traI	45287	47509	reverse	KC964605	100.00%	pALTS27
traH	45601	45963	reverse	KC964605	100.00%	pALTS27
traJ	47541	47915	reverse	KC964605	100.00%	pALTS27
traK	48255	48644	forward	KC964605	100.00%	pALTS27
traL	48644	49369	forward	KC964605	100.00%	pALTS27
traM	49369	49809	forward	KC964605	100.00%	pALTS27
hypothetical_protein	49859	50470	reverse	KC964605	100.00%	pALTS27
hypothetical_protein	50496	50843	reverse	KC964605	100.00%	pALTS27
kfrA	51012	51953	reverse	KC964605	100.00%	pALTS27
korB	52140	53183	reverse	KC964605	100.00%	pALTS27
incC1	53180	54256	reverse	KC964605	100.00%	pALTS27
incC2	53180	53944	reverse	KC964605	100.00%	pALTS27
korA	53941	54252	reverse	KC964605	100.00%	pALTS27
kleF	54348	54461	reverse	KC964605	100.00%	pALTS27
kleF	54413	54655	reverse	KC964605	100.00%	pALTS27
kleE	54657	54989	reverse	KC964605	100.00%	pALTS27
kleB	55105	55323	reverse	KC964605	100.00%	pALTS27
kleA	55384	55659	reverse	KC964605	100.00%	pALTS27
korC	55770	56024	reverse	KC964605	100.00%	pALTS27
klcB	56021	57055	reverse	KC964605	100.00%	pALTS27
klcA	57096	57530	reverse	KC964605	100.00%	pALTS27
pALTS28:						
hypothetical_protein	1	1560	reverse	FJ666348	96.00%	pALTS28
hypothetical_protein	1472	1732	reverse	FJ666348	98.08%	pALTS28
hypothetical_protein	1768	2748	reverse	YP_001672070.1	89.79%	pALTS28
hypothetical_protein	2723	3094	reverse	YP_001672069.1	99.15%	pALTS28
hypothetical_protein	3106	3957	forward	YP_001672068.1	97.62%	pALTS28
hypothetical_protein	4034	4273	reverse	FJ666348	98.33%	pALTS28
hypothetical_protein	4299	4676	reverse	YP_009067979.1	28.07%	pALTS28
hypothetical_protein	4687	4896	reverse	FJ666348	94.76%	pALTS28
hypothetical_protein	4897	5457	forward	FJ666348	96.80%	pALTS28
hypothetical_protein	5623	6051	reverse	YP_001672064.1	96.48%	pALTS28
hypothetical_protein	6061	6297	reverse	FJ666348	94.94%	pALTS28
hypothetical_protein	6354	6494	reverse	FJ666348	94.33%	pALTS28

hypothetical_protein	6582	6848	reverse	YP_001672061.1	95.46%	pALTS28
hypothetical_protein	6957	7505	reverse	FJ666348	100.00%	pALTS28
hypothetical_protein	7526	7945	reverse	FJ666348	99.05%	pALTS28
hypothetical_protein	8920	9315	forward	FJ666348	99.75%	pALTS28
traA	9373	10134	forward	FJ666348	99.34%	pALTS28
traB	10144	12459	forward	FJ666348	99.57%	pALTS28
traC	12624	12932	forward	FJ666348	100.00%	pALTS28
traD	12942	13265	forward	FJ666348	100.00%	pALTS28
traE	13272	15752	forward	FJ666348	99.52%	pALTS28
traF	15758	16480	forward	FJ666348	99.86%	pALTS28
traG	16584	16880	forward	FJ666348	99.66%	pALTS28
*traH**	16891	17172	forward	FJ666348	100.00%	pALTS28
traH	17176	18258	forward	FJ666348	99.35%	pALTS28
traI	18395	18559	forward	FJ666348	100.00%	pALTS28
traJ	18565	19275	forward	FJ666348	99.58%	pALTS28
traK	19272	20144	forward	FJ666348	98.74%	pALTS28
traL	20144	21304	forward	FJ666348	99.40%	pALTS28
traM	21288	22355	forward	FJ666348	99.81%	pALTS28
hypothetical_protein	22361	22894	forward	FJ666348	93.79%	pALTS28
traN	22872	25376	forward	FJ666348	99.68%	pALTS28
traO'	25477	26172	forward	FJ666348	99.86%	pALTS28
traO	26184	28376	forward	FJ666348	98.46%	pALTS28
hypothetical_protein	28197	28376	forward	FJ666348	97.22%	pALTS28
traP	28373	28783	forward	FJ666348	98.05%	pALTS28
hypothetical_protein	29175	29735	forward	FJ666348	95.01%	pALTS28
hypothetical_protein	29749	30327	forward	FJ666348	96.72%	pALTS28
hypothetical_protein	30416	30895	forward	FJ666348	96.67%	pALTS28
traS	30950	32056	reverse	FJ666348	96.93%	pALTS28
hypothetical_protein	32041	32580	reverse	FJ666348	98.16%	pALTS28
hypothetical_protein	32847	33317	reverse	FJ666348	95.75%	pALTS28
korB	33320	34465	reverse	YP_001672036.1	99.21%	pALTS28
incC	34466	35080	reverse	YP_001672035.1	99.51%	pALTS28
hypothetical_protein	35254	35631	reverse	FJ666348	95.50%	pALTS28
hypothetical_protein	35656	36189	reverse	YP_001672033.1	97.00%	pALTS28
hypothetical_protein	36585	36749	reverse	YP_006960845.1	55.56%	pALTS28
hypothetical_protein	36904	37095	reverse	YP_006963948.1	66.67%	pALTS28
kfrA	37583	38617	reverse	FJ666348	98.65%	pALTS28
hypothetical_protein	38720	39016	reverse	YP_008110933.1	29.00%	pALTS28
hypothetical_protein	39058	39450	reverse	FJ666348	99.49%	pALTS28
hypothetical_protein	39454	39636	reverse	YP_001672030.1	100.00%	pALTS28
hypothetical_protein	39718	41184	reverse	YP_001672029.1	95.00%	pALTS28
aph(6)-Id	42024	42860	reverse	YP_008998863.1	99.64%	pALTS28
aph(3'')-Ib	42860	43648	reverse	YP_008167029.1	100.00%	pALTS28

hypothetical_protein	43808	44602	forward	YP_006956129.1	67.00%	pALTS28
hypothetical_protein	44605	45054	forward	YP_001312840.1	67.27%	pALTS28
hypothetical_protein	45145	45981	forward	YP_001312841.1	58.67%	pALTS28
hypothetical_protein	46219	46530	forward	YP_001314256.1	41.94%	pALTS28
tet(X)	46554	47690	forward	YP_006961366.1	85.00%	pALTS28
hypothetical_protein	47708	48568	forward	YP_004810312.1	85.56%	pALTS28
hypothetical_protein	48917	49453	forward	YP_006964765.1	22.58%	pALTS28
glmM	49742	50044	reverse	YP_002791823.1	100.00%	pALTS28
sul2	50131	50946	reverse	YP_002650687.1	100.00%	pALTS28
hypothetical_protein	51343	51600	forward	YP_008998087.1	38.30%	pALTS28
mph(E)	51739	52623	reverse	YP_006952444.1	100.00%	pALTS28
msr(E)	52679	54154	reverse	YP_724476.1	100.00%	pALTS28
aph(6)-Id	55085	55921	reverse	YP_008998863.1	99.64%	pALTS28
aph(3'')-Ib	55921	56739	reverse	YP_008167029.1	99.63%	pALTS28
tnpR	56790	57404	reverse	YP_006960710.1	100.00%	pALTS28
tnpA	57530	60415	forward	YP_006960758.1	100.00%	pALTS28
hypothetical_protein	60448	1	reverse	YP_001672028.1	93.51%	pALTS28
pALTS29:						
trfA	1	1221	reverse	AJ639924	100.00%	pALTS29
ssb	1269	1610	reverse	AJ639924	100.00%	pALTS29
trbA	1724	2086	forward	AJ639924	100.00%	pALTS29
trbB	2394	3359	forward	AJ639924	100.00%	pALTS29
trbC	3373	3837	forward	AJ639924	100.00%	pALTS29
trbD	3841	4152	forward	AJ639924	100.00%	pALTS29
trbE	4149	6707	forward	AJ639924	100.00%	pALTS29
trbF	6704	7486	forward	AJ639924	100.00%	pALTS29
trbG	7504	8403	forward	AJ639924	100.00%	pALTS29
trbH	8400	8894	forward	AJ639924	100.00%	pALTS29
trbI	8899	10320	forward	AJ639924	100.00%	pALTS29
trbJ	10341	11105	forward	AJ639924	100.00%	pALTS29
trbK	11115	11342	forward	AJ639924	100.00%	pALTS29
trbL	11353	13083	forward	AJ639924	100.00%	pALTS29
trbM	13101	13688	forward	AJ639924	100.00%	pALTS29
trbN	13702	14337	forward	AJ639924	100.00%	pALTS29
trbO	14366	14632	forward	AJ639924	100.00%	pALTS29
trbP	14632	15330	forward	AJ639924	100.00%	pALTS29
upf30.5	15346	15777	forward	AJ639924	100.00%	pALTS29
upf31.0	15932	16606	forward	AJ639924	99.85%	pALTS29
parA	16608	17267	reverse	AJ639924	100.00%	pALTS29
blaOXA	17717	18499	forward	AJ639924	100.00%	pALTS29
tniC	18594	19217	reverse	AJ639924	100.00%	pALTS29
'intI1	19579	19800	reverse	AJ639924	100.00%	pALTS29
tnpA	19866	20570	forward	AJ639924	100.00%	pALTS29

msr(E)	21020	22495	forward	YP_724476.1	100.00%	pALTS29
mph(E)	22551	23435	forward	YP_006952444.1	100.00%	pALTS29
tnpA	23519	24223	forward	AJ639924	100.00%	pALTS29
intI1'	24276	25073	reverse	AJ639924	100.00%	pALTS29
cmlA1	25416	26675	forward	AJ639924	100.00%	pALTS29
hypothetical_protein	26867	27214	forward	AJ639924	100.00%	pALTS29
qacE	26867	27214	forward	AJ639924	100.00%	pALTS29
sul1	27208	28047	forward	AJ639924	100.00%	pALTS29
N-acetyltransferase	28175	28675	forward	YP_002891165.1	100.00%	pALTS29
tnpA	29182	29946	forward	AJ639924	100.00%	pALTS29
traC	30568	34914	reverse	AJ639924	100.00%	pALTS29
traD	34918	35307	reverse	AJ639924	100.00%	pALTS29
traE	35329	37392	reverse	AJ639924	100.00%	pALTS29
traF	37405	37941	reverse	AJ639924	100.00%	pALTS29
traG	37938	39851	reverse	AJ639924	100.00%	pALTS29
traI	39848	42088	reverse	AJ639924	100.00%	pALTS29
traH	40150	40542	reverse	AJ639924	100.00%	pALTS29
traJ	42123	42497	reverse	AJ639924	100.00%	pALTS29
traK	42872	43270	forward	AJ639924	100.00%	pALTS29
traL	43270	43995	forward	AJ639924	100.00%	pALTS29
traM	43995	44435	forward	AJ639924	100.00%	pALTS29
traN	44638	45297	reverse	AJ639924	100.00%	pALTS29
traO	45320	45667	reverse	AJ639924	100.00%	pALTS29
kfrA	45839	46870	reverse	AJ639924	100.00%	pALTS29
korB	47049	48098	reverse	AJ639924	100.00%	pALTS29
incC1	48095	49171	reverse	AJ639924	100.00%	pALTS29
incC2	48095	48859	reverse	AJ639924	100.00%	pALTS29
korA	48856	49158	reverse	AJ639924	100.00%	pALTS29
kleF	49272	49802	reverse	AJ639924	100.00%	pALTS29
kleE	49804	50190	reverse	AJ639924	100.00%	pALTS29
kleB	50278	50493	reverse	AJ639924	100.00%	pALTS29
kleA	50552	50788	reverse	AJ639924	100.00%	pALTS29
klcC	50946	51203	reverse	AJ639924	100.00%	pALTS29
klcB	51220	52428	reverse	AJ639924	100.00%	pALTS29
klcA	52480	52908	reverse	AJ639924	100.00%	pALTS29
pALTS31:						
trfA	1	1221	reverse	KC170279	100.00%	pALTS31
ssb	1268	1609	reverse	KC170279	100.00%	pALTS31
trbA	1723	2085	forward	KC170279	100.00%	pALTS31
trbB	2395	3357	forward	KC170279	100.00%	pALTS31
trbC	3374	3838	forward	KC170279	100.00%	pALTS31
trbD	3842	4153	forward	KC170279	100.00%	pALTS31
trbE	4150	6708	forward	KC170279	100.00%	pALTS31

trbF	6705	7487	forward	KC170279	100.00%	pALTS31
trbG	7505	8404	forward	KC170279	100.00%	pALTS31
trbH	8407	8895	forward	KC170279	100.00%	pALTS31
trbI	8900	10321	forward	KC170279	100.00%	pALTS31
trbJ	10342	11106	forward	KC170279	100.00%	pALTS31
trbK	11116	11343	forward	KC170279	100.00%	pALTS31
trbL	11360	13072	forward	KC170279	100.00%	pALTS31
trbM	13090	13677	forward	KC170279	100.00%	pALTS31
trbN	13691	14326	forward	KC170279	100.00%	pALTS31
trbO	14355	14621	forward	KC170279	99.63%	pALTS31
trbP	14621	15319	forward	KC170279	100.00%	pALTS31
upf30.5	15335	15766	forward	KC170279	100.00%	pALTS31
hypothetical_protein	15738	16418	forward	YP_007878480.1	96.58%	pALTS31
hypothetical_protein	16504	16884	forward	YP_001966049.1	34.43%	pALTS31
traC	19173	23519	reverse	KC170279	100.00%	pALTS31
traD	23523	23912	reverse	KC170279	100.00%	pALTS31
traE	23934	25997	reverse	KC170279	100.00%	pALTS31
traF	26009	26545	reverse	KC170279	100.00%	pALTS31
traG	26542	28455	reverse	KC170279	100.00%	pALTS31
traI	28452	30692	reverse	KC170279	100.00%	pALTS31
traH	28754	29146	reverse	KC170279	100.00%	pALTS31
traJ	30727	31101	reverse	KC170279	100.00%	pALTS31
traK	31475	31873	forward	KC170279	100.00%	pALTS31
traL	31873	32598	forward	KC170279	100.00%	pALTS31
traM	32598	33038	forward	KC170279	100.00%	pALTS31
kfrC	33241	33894	reverse	KC170279	100.00%	pALTS31
kfrB	33923	34270	reverse	KC170279	99.71%	pALTS31
kfrA	34441	35472	reverse	KC170279	100.00%	pALTS31
korB	35652	36701	reverse	KC170279	100.00%	pALTS31
incC1	36698	37774	reverse	KC170279	100.00%	pALTS31
korA	37459	37761	reverse	KC170279	100.00%	pALTS31
kleF	37875	38405	reverse	KC170279	100.00%	pALTS31
kleE	38407	38736	reverse	KC170279	100.00%	pALTS31
kleB	38881	39096	reverse	KC170279	100.00%	pALTS31
kleA	39155	39391	reverse	KC170279	100.00%	pALTS31
korC	39551	39808	reverse	KC170279	100.00%	pALTS31
klcB	39825	41030	reverse	KC170279	99.90%	pALTS31
klcA	41082	41510	reverse	KC170279	100.00%	pALTS31
kluA	41679	41948	forward	KC170279	100.00%	pALTS31
kluB	41945	42259	forward	KC170279	100.00%	pALTS31
tn3 resolvase	43364	43921	reverse	Isfinder-ISPa38	100.00%	pALTS31
hypothetical_protein	43915	44286	reverse	Isfinder-ISPa38	100.00%	pALTS31
hypothetical_protein	44283	44783	reverse	Isfinder-ISPa38	100.00%	pALTS31

hypothetical_protein	44780	45106	reverse	Isfinder-ISPa38	100.00%	pALTS31
hypothetical_protein	45361	45735	reverse	Isfinder-ISPa38	100.00%	pALTS31
hypothetical_protein	45957	4634	reverse	Isfinder-ISPa38	100.00%	pALTS31
intl1	46911	47924	reverse	YP_002286930.1	100.00%	pALTS31
hypothetical_protein	48082	48555	forward	YP_006953993.1	81.53%	pALTS31
hypothetical_protein	48751	49974	forward	YP_006958744.1	61.77%	pALTS31
aadA1	50082	50873	forward	YP_003864165.1	99.62%	pALTS31
blaOXA-2	50929	51777	forward	YP_006953608.1	100.00%	pALTS31
qacE	51913	52260	forward	YP_002791712.1	100.00%	pALTS31
sul1	52254	53093	forward	YP_008725247.1	99.65%	pALTS31
n-acetyltransferase	53221	53721	forward	YP_002891165.1	100.00%	pALTS31
tnpA	54192	54992	forward	YP_003108355.1	99.62%	pALTS31
hypothetical_protein	55011	55166	reverse	YP_003675799.1	73.91%	pALTS31
tnpA	55281	58247	forward	YP_007969442.1	100.00%	pALTS31
pALTS32:						
trfA	1	1224	reverse	KC964605	100.00%	pALTS32
ssb	1275	1616	reverse	KC964605	100.00%	pALTS32
trbA	1728	2102	forward	KC964605	100.00%	pALTS32
trbB	2392	3354	forward	KC964605	100.00%	pALTS32
trbC	3368	3832	forward	KC964605	100.00%	pALTS32
trbD	3836	4147	forward	KC964605	100.00%	pALTS32
trbE	4144	6702	forward	KC964605	100.00%	pALTS32
trbF	6699	7472	forward	KC964605	100.00%	pALTS32
trbG	7490	8383	forward	KC964605	100.00%	pALTS32
trbH	8386	8841	forward	KC964605	100.00%	pALTS32
trbI	8845	10248	forward	KC964605	100.00%	pALTS32
trbJ	10267	11037	forward	KC964605	100.00%	pALTS32
trbK	11047	11220	forward	KC964605	100.00%	pALTS32
trbK	11217	11432	forward	KC964605	100.00%	pALTS32
trbL	11444	13360	forward	KC964605	100.00%	pALTS32
trbM	13396	13983	forward	KC964605	100.00%	pALTS32
trbN	14000	14629	forward	KC964605	100.00%	pALTS32
trbO	14658	14927	forward	KC964605	100.00%	pALTS32
trbP	14924	15622	forward	KC964605	100.00%	pALTS32
upf30.5	15637	16068	forward	KC964605	100.00%	pALTS32
hypothetical_protein	16111	16524	forward	KC964605	100.00%	pALTS32
hypothetical_protein	16550	17518	forward	KC964605	100.00%	pALTS32
parA	17501	18106	reverse	KC964605	100.00%	pALTS32
hypothetical_protein	18282	18818	reverse	KC964605	100.00%	pALTS32
hypothetical_protein	18830	19225	reverse	KC964605	100.00%	pALTS32
hypothetical_protein	19222	19473	reverse	KC964605	100.00%	pALTS32
hypothetical_protein	19655	20221	forward	KC964605	100.00%	pALTS32
intI1	20569	20784	reverse	KC964605	100.00%	pALTS32

tnpA	20844	21560	forward	KC964605	100.00%	pALTS32
msrE	22010	23485	forward	KC964605	100.00%	pALTS32
mphE	23541	24425	forward	KC964605	100.00%	pALTS32
tnpA	24497	25213	forward	KC964605	100.00%	pALTS32
intI1	25274	26063	reverse	KC964605	100.00%	pALTS32
qacL	26314	26646	forward	KC964605	100.00%	pALTS32
qacEdelta1	26819	27166	forward	KC964605	100.00%	pALTS32
sul1	27160	27999	forward	KC964605	100.00%	pALTS32
hypothetical_protein	28127	28627	forward	KC964605	100.00%	pALTS32
istB	28803	29585	reverse	KC964605	100.00%	pALTS32
istA	29575	31089	reverse	KC964605	100.00%	pALTS32
tetR	31931	32608	reverse	KC964605	100.00%	pALTS32
tetA	32687	33886	forward	KC964605	100.00%	pALTS32
hypothetical_protein	33918	34778	reverse	KC964605	100.00%	pALTS32
tniA	34763	35470	reverse	KC964605	100.00%	pALTS32
traC	35769	39476	reverse	KC964605	100.00%	pALTS32
traD	39480	39863	reverse	KC964605	100.00%	pALTS32
traE	39868	41943	reverse	KC964605	100.00%	pALTS32
traF	41958	42494	reverse	KC964605	100.00%	pALTS32
traG	42491	44395	reverse	KC964605	100.00%	pALTS32
traI	44392	46614	reverse	KC964605	100.00%	pALTS32
traH	44706	45068	reverse	KC964605	100.00%	pALTS32
traJ	46646	47020	reverse	KC964605	100.00%	pALTS32
traK	47360	47749	forward	KC964605	100.00%	pALTS32
traL	47749	48474	forward	KC964605	100.00%	pALTS32
traM	48474	48914	forward	KC964605	100.00%	pALTS32
hypothetical_protein	48964	49575	reverse	KC964605	100.00%	pALTS32
hypothetical_protein	49601	49948	reverse	KC964605	100.00%	pALTS32
kfrA	50117	51058	reverse	KC964605	100.00%	pALTS32
korB	51245	52288	reverse	KC964605	100.00%	pALTS32
incC1	52285	53361	reverse	KC964605	100.00%	pALTS32
incC2	52285	53049	reverse	KC964605	100.00%	pALTS32
korA	53046	53357	reverse	KC964605	100.00%	pALTS32
kleF	53453	53566	reverse	KC964605	100.00%	pALTS32
kleF	53518	53760	reverse	KC964605	100.00%	pALTS32
kleE	53762	54094	reverse	KC964605	100.00%	pALTS32
kleB	54210	54428	reverse	KC964605	100.00%	pALTS32
kleA	54489	54764	reverse	KC964605	100.00%	pALTS32
korC	54875	55129	reverse	KC964605	100.00%	pALTS32
klcB	55126	56160	reverse	KC964605	100.00%	pALTS32
klcA	56201	56635	reverse	KC964605	100.00%	pALTS32
pALTS33:						
trfA	1	1224	reverse	YP_006965454.1	98.28%	pALTS33

ssb	1272	1610	reverse	JX469829	100.00%	pALTS33
trbA	1720	2079	forward	YP_006965456.1	99.16%	pALTS33
hypothetical_protein	2347	3357	forward	YP_006965457.1	98.44%	pALTS33
trbC	3379	3843	forward	JX469829	99.14%	pALTS33
trbD	3847	4158	forward	JX469829	100.00%	pALTS33
trbE	4155	6713	forward	JX469829	99.30%	pALTS33
trbF	6710	7498	forward	JX469829	98.99%	pALTS33
trbG	7480	8409	forward	YP_302640.1	99.01%	pALTS33
trbH	8412	8873	forward	JX469829	98.92%	pALTS33
hypothetical_protein	8839	10257	forward	YP_006965464.1	94.82%	pALTS33
hypothetical_protein	10247	11047	forward	YP_302643.1	99.22%	pALTS33
trbK	11057	11275	forward	JX469829	100.00%	pALTS33
hypothetical_protein	11287	13011	forward	YP_006965467.1	98.91%	pALTS33
trbM	13021	13608	forward	YP_001967682.1	98.44%	pALTS33
trbN	13610	14245	forward	YP_006965469.1	99.53%	pALTS33
hypothetical_protein	14274	14543	forward	YP_302648.1	96.63%	pALTS33
trbP	14846	15238	forward	YP_302649.1	99.23%	pALTS33
hypothetical_protein	15254	15697	forward	YP_006965472.1	98.29%	pALTS33
upf31.0	15889	16071	forward	YP_302606.1	98.33%	pALTS33
tnpA	16081	17289	reverse	YP_001101847.1	100.00%	pALTS33
hypothetical_protein	17341	17901	forward	YP_302606.1	94.38%	pALTS33
hypothetical_protein	17957	18505	forward	JX469829	98.18%	pALTS33
hypothetical_protein	18462	19352	reverse	YP_006965475.1	98.08%	pALTS33
hypothetical_protein	19357	19683	reverse	YP_308723.1	32.00%	pALTS33
intI1	19936	20949	reverse	YP_002286930.1	100.00%	pALTS33
hypothetical_protein	20464	20859	forward	YP_009023530.1	99.23%	pALTS33
blaOXA-2	21107	21934	forward	YP_006953608.1	100.00%	pALTS33
aadA1	21971	22762	forward	YP_003864165.1	99.62%	pALTS33
qacE	22926	23273	forward	YP_001552096.1	100.00%	pALTS33
sul1	23267	24106	forward	YP_002791829.1	99.35%	pALTS33
n-acetyltransferase	24234	24734	forward	YP_002891165.1	100.00%	pALTS33
hypothetical_protein	24717	24860	reverse	YP_006955149.1	100.00%	pALTS33
tnpA	25040	25840	forward	YP_003108355.1	99.62%	pALTS33
pin	26313	26996	reverse	YP_008508472.1	100.00%	pALTS33
hypothetical_protein	27043	27312	forward	YP_245466.1	100.00%	pALTS33
hypothetical_protein	27309	27704	forward	YP_245465.1	100.00%	pALTS33
hypothetical_protein	27701	28252	forward	YP_007509605.1	99.45%	pALTS33
tnpA	28488	31454	forward	YP_007969442.1	94.03%	pALTS33
relE	32727	33008	reverse	YP_005351605.1	98.93%	pALTS33
relB	32995	33258	reverse	JX469829	100.00%	pALTS33
hypothetical_protein	33384	33860	forward	YP_006965499.1	75.17%	pALTS33
hypothetical_protein	33860	34123	forward	YP_007878456.1	95.40%	pALTS33
hypothetical_protein	34120	35337	forward	YP_006965497.1	97.50%	pALTS33

hypothetical_protein	35306	35611	forward	YP_001967658.1	81.18%	pALTS33
kleA	35767	36003	forward	JX469829	98.31%	pALTS33
kleE	36212	36538	forward	JX469829	99.39%	pALTS33
kleF	36540	36869	forward	JX469829	100.00%	pALTS33
incC1	36963	38045	forward	JX469829	99.82%	pALTS33
korA	36976	37284	forward	JX469829	100.00%	pALTS33
korB	38042	39109	forward	JX469829	99.53%	pALTS33
kfrA	39311	40312	forward	JX469829	99.30%	pALTS33
hypothetical_protein	40440	40787	forward	YP_001967649.1	96.52%	pALTS33
kfrC	40817	41455	forward	JX469829	99.53%	pALTS33
traM	41651	42091	reverse	JX469829	99.09%	pALTS33
traL	42091	42816	reverse	YP_006965484.1	100.00%	pALTS33
traK	42816	43235	reverse	YP_302618.1	98.56%	pALTS33
hypothetical_protein	43598	43819	forward	YP_001967649.1	96.52%	pALTS33
traJ	43824	44195	forward	JX469829	99.46%	pALTS33
traI	44230	46488	forward	JX469829	99.47%	pALTS33
traH	45776	46186	forward	JX469829	99.51%	pALTS33
traG	46485	48389	forward	JX469829	99.32%	pALTS33
traF	48386	48922	forward	JX469829	99.63%	pALTS33
traE	48937	51000	forward	JX469829	99.56%	pALTS33
traD	51025	51417	forward	JX469829	99.75%	pALTS33
hypothetical_protein	51421	56397	forward	YP_006965476.1	99.02%	pALTS33
hypothetical_protein	56756	58456	reverse	YP_001314866.1	40.51%	pALTS33
hypothetical_protein	58458	58820	reverse	YP_008004824.1	51.75%	pALTS33
hypothetical_protein	58820	59137	reverse	NP_396299.1	28.00%	pALTS33
hypothetical_protein	59435	60640	forward	YP_003108355.1	99.62%	pALTS33
hypothetical_protein	60666	61223	forward	YP_245470.1	85.17%	pALTS33
hypothetical_protein	61347	63722	reverse	NP_862419.1	34.18%	pALTS33
hypothetical_protein	63863	64267	reverse	YP_006965120.1	24.44%	pALTS33
hypothetical_protein	64264	65385	reverse	YP_006965510.1	72.50%	pALTS33
hypothetical_protein	65297	68218	reverse	YP_709286.1	80.46%	pALTS33
hypothetical_protein	68221	68718	reverse	YP_161723.1	92.07%	pALTS33
hypothetical_protein	68994	69272	reverse	XP_022467370.1	33.33%	pALTS33
hypothetical_protein	69316	69738	forward	NP_052408.1	56.52%	pALTS33
hypothetical_protein	69894	32	forward	YP_195911.1	89.89%	pALTS33

Genes annotated on the plasmid are listed with a source reference and relative identities to the source. For annotations transferred from the most similar plasmid, the accession for the plasmid is listed. For annotations transferred from the most similar gene, the accession for the gene is listed. For those identified from ISfinder, the ISfinder IS element name is listed.

SUPPLEMENTAL REFERENCES

Langmead, B., and Salzberg, S. L. (2012). Fast gapped-read alignment with Bowtie 2. *Nature Methods* 9, 357–359. doi:10.1038/nmeth.1923.

Nurk, S., Bankevich, A., Antipov, D., Gurevich, A., Korobeynikov, A., Lapidus, A., et al. (2013). Assembling Genomes and Mini-metagenomes from Highly Chimeric Reads. in *Research in Computational Molecular Biology* Lecture Notes in Computer Science., eds. M. Deng, R. Jiang, F. Sun, and X. Zhang (Berlin, Heidelberg: Springer), 158–170. doi:10.1007/978-3-642-37195-0_13.

Wick, R. R., Judd, L. M., Gorrie, C. L., and Holt, K. E. (2017). Unicycler: Resolving bacterial genome assemblies from short and long sequencing reads. *PLOS Computational Biology* 13, e1005595. doi:10.1371/journal.pcbi.1005595.
